# Inner layer security reinforcement for instant payment systems: a dual layer encryption-steganography evaluation in Brunei's digital payment context

**DOI:** 10.3389/fdata.2026.1826953

**Published:** 2026-06-17

**Authors:** Ampuan Shazani bin Ampuan Haji Sadikin, Heru Susanto

**Affiliations:** 1 Science in Business and Technology Management, Universiti Teknologi Brunei, Bandar Seri Begawan, Brunei; 2 School of Business, Universiti Teknologi Brunei, Bandar Seri Begawan, Brunei; 3 Center for Research Collaboration Theory Graph and Combinatory, UTB School of Business, Bandar Seri Begawan, Brunei; 4 National Research and Innovation Agency, The Indonesia Institute of Science, Jakarta, Indonesia; 5 Department of Mathematics, University of Indonesia, Depok, Indonesia; 6 Department of Mathematics, Bandung Institute of Technology, Bandung, Indonesia; 7 Department of Computer Science, Tunghai University, Taichung, Taiwan

**Keywords:** AES encryption, digital payments, human factor risk, spread spectrum steganography, zero trust

## Abstract

**Introduction:**

The rapid adoption of real-time digital payment systems introduces cybersecurity risks that extend beyond technical vulnerabilities to include significant human and organizational factors.

**Methods:**

This study evaluates a dual-layer data protection mechanism combining AES-128 encryption with spread spectrum audio steganography within Brunei's digital payment context. Stakeholder interviews with Cyber Security Brunei (CSB), National Digital Payments Network (NDPx), and a local bank were conducted alongside experimental testing using Stripe Sandbox.

**Results:**

Human factor issues accounted for 47% of identified cybersecurity concerns. Experimental results demonstrated that spread spectrum steganography achieved an 87.5% robustness rate across eight attack scenarios while maintaining near real-time performance with an average processing time of 568.82 ms and acceptable audio quality (PSNR 26.30 dB). Sandbox validation confirmed feasibility within realistic payment workflows.

**Discussion:**

The findings support data-centric security as a compensating control in human-dominated threat environments and demonstrate the viability of combining encryption and steganography to reinforce instant payment security.

## 1 Introduction

The expansion of real-time digital payment systems has transformed financial transactions by improving speed, accessibility, and financial inclusion (World Bank, 2022). In Brunei Darussalam, the introduction of TARUS as a national instant payment platform represents a major step toward a cashless economy aligned with Wawasan Brunei 2035 (Ministry of Finance and Economy (MOFE), 2019). However, this transformation also amplifies cybersecurity risks, particularly those arising from human behavior rather than system failure. Insights from national stakeholders highlight that phishing, APP fraud, and Otp-based scams dominate the current threat landscape. Interviews with CSB, NDPx, and a

participating bank indicate that nearly half of identified cybersecurity incidents are enabled through user manipulation, where transactions are legitimately authorized but maliciously induced (Cyber Security Brunei (CSB), 2024). This pattern reflects a critical limitation of traditional perimeter-based security, where once a user is deceived into approving a transaction, cryptographic channels and authentication mechanisms offer little protection (Hadnagy, 2018; Mitnick and Simon, 2002).

While strengthening awareness, governance and regulation remains essential, such measures cannot fully eliminate human error in high speed payment environments. Instant payment systems execute transactions immediately, leaving minimal opportunity for reversal or manual intervention (Arner et al., 2024). This creates a gap between behavioral risk exposure and technical safeguards, particularly at the data and metadata level. This research addresses that gap by proposing and evaluating a dual layer protection mechanism that combines AES encryption with audio steganography. Rather than attempting to prevent social engineering directly, the approach focuses on limiting the impact of human driven compromise by concealing and protecting sensitive transaction data even after authorization. By positioning encryption-steganography as an inner defensive layer aligned with Defense in Depth (DiD) and Zero Trust Architecture (ZTA) principles, this study demonstrates how technical controls can meaningfully support resilience in a sociotechnical threat environment (National Institute of Standards and Technology (NIST), 2020; Pfleeger et al., 2015).

The contributions of this paper are threefold. First, it empirically characterizes cybersecurity risk in Brunei's digital payment ecosystem using stakeholder interviews and policy analysis, revealing that 47% of risks originate from human factors. Second, it experimentally evaluates an audio steganography technique integrated with encryption under performance, quality, and robustness metrics, demonstrating Spread Spectrum (SS) superiority for payment security application. Third, it demonstrates how data-centric protection can function as a compensating control aligned with DiD and ZTA principles in socio-technical threat environments.

## 2 Theoretical framework and literature review

This research is supported by three theoretical frameworks that guide the analysis and design of security mechanisms. The CIA Triad (Confidentiality, Integrity, and Availability) remains a core model for assessing information system security (Mitchell et al., 2023). Confidentiality relates to restricting access to sensitive information, integrity concerns ensuring that data remains unmodified and trustworthy, and availability refers to keeping systems and services accessible whenever needed (Tripathi, 2025).

Within this research, AES encryption contributes to confidentiality and integrity by protecting the content of transaction data, while steganography adds another layer of confidentiality by concealing the presence of the data itself. DiD emphasizes that effective cybersecurity cannot rely on a single control; instead, multiple overlapping safeguards must operate together to resist or slow down attacks (Pfleeger et al., 2015). Academic literature recommends layering several independent methods for systems that handle high value transactions (Abdelghani, 2019). The combination of AES encryption and audio steganography implemented in this research exemplifies an inner layer DiD mechanism, offering additional protection even if outer layers fail.

ZTA redefines security around constant validation, restricted privilege, and the premise that a breach may already exist within the system (Kindervag, 2010; National Institute of Standards and Technology (NIST), 2020). ZTA explicitly rejects implicit trust in users or endpoints, making it particularly applicable to digital payment infrastructures where social engineering attacks succeed by exploiting trust assumptions (Nasiruzzaman et al., 2025). Socio-technical resilience encompasses the capacity of institutions and users to sustain secure operations by strengthening human behavior, refining processes, implementing layered security mechanisms, and cultivating organizational learning (Patriarca et al., 2021). This conceptualization recognizes that vulnerabilities extend beyond software bugs to include human actions, organizational routines, and procedural shortcomings (Furnell and Shah, 2020). Together, these frameworks justify the dual layer approach, where CIA demonstrates why confidentiality breaches require both encryption and concealment, DiD shows that single layer controls are insufficient against socially engineered attacks, and ZTA confirms that hidden encrypted payloads support least privilege data handling even when trust assumptions fail.

Encryption remains a cornerstone of securing transaction data. Symmetric key algorithms such as AES are widely adopted due to their high performance and strong resistance to attacks (Akash, 2025; Daemen and Rijmen, 2001). Modern payment infrastructures frequently adopt AESGCM because it provides authenticated encryption, ensuring confidentiality while simultaneously verifying integrity (McGrew and Viega, 2021; National Institute of Standards and Technology (NIST), 2024). However, encryption alone does not prevent adversaries from analyzing communication metadata or identifying sensitive traffic patterns (Sasy and Goldberg, 2024).

Research on covert communication techniques indicates that pairing encryption with steganography can offer an additional layer of concealment by hiding the presence of encrypted data (Singh et al., 2021; Radivilova et al., 2025). Steganography hides the existence of secret messages inside innocuous carriers. Common audio steganography techniques include SS, which distributes the embedded payload across a wide range of audio samples using pseudo random sequences, enhancing resistance to noise, filtering, and quantization effects while trading capacity and perceptual quality for resilience (Gupta, 1997; Marvel et al., 1999). Literature consistently reports a trade off triangle, i.e., capacity-imperceptibility-robustness (Cox et al., 2007), with recent robust frameworks embedding encrypted payloads with error correction coding to improve robustness (Wu, 2018; Wang et al., 2022).

While many studies evaluate AES and steganography in laboratory settings, few situate the techniques within operational deployment contexts and examine policy, human, and system integration constraints that affect adoptability. This research addresses this gap by contextualizing hybrid techniques within Brunei's national digital payment architecture and conducting comparative evaluation under payment relevant transformations, benchmarking SS with AES payloads and Reed-Solomon error correction to evaluate viability in payment flows. Previous studies have also examined the relationship between encryption reliability, steganography, and error correction in secure communication systems (Li et al., 2011; Cohen et al., 2022).

## 3 Methodology

**Figure 1:**
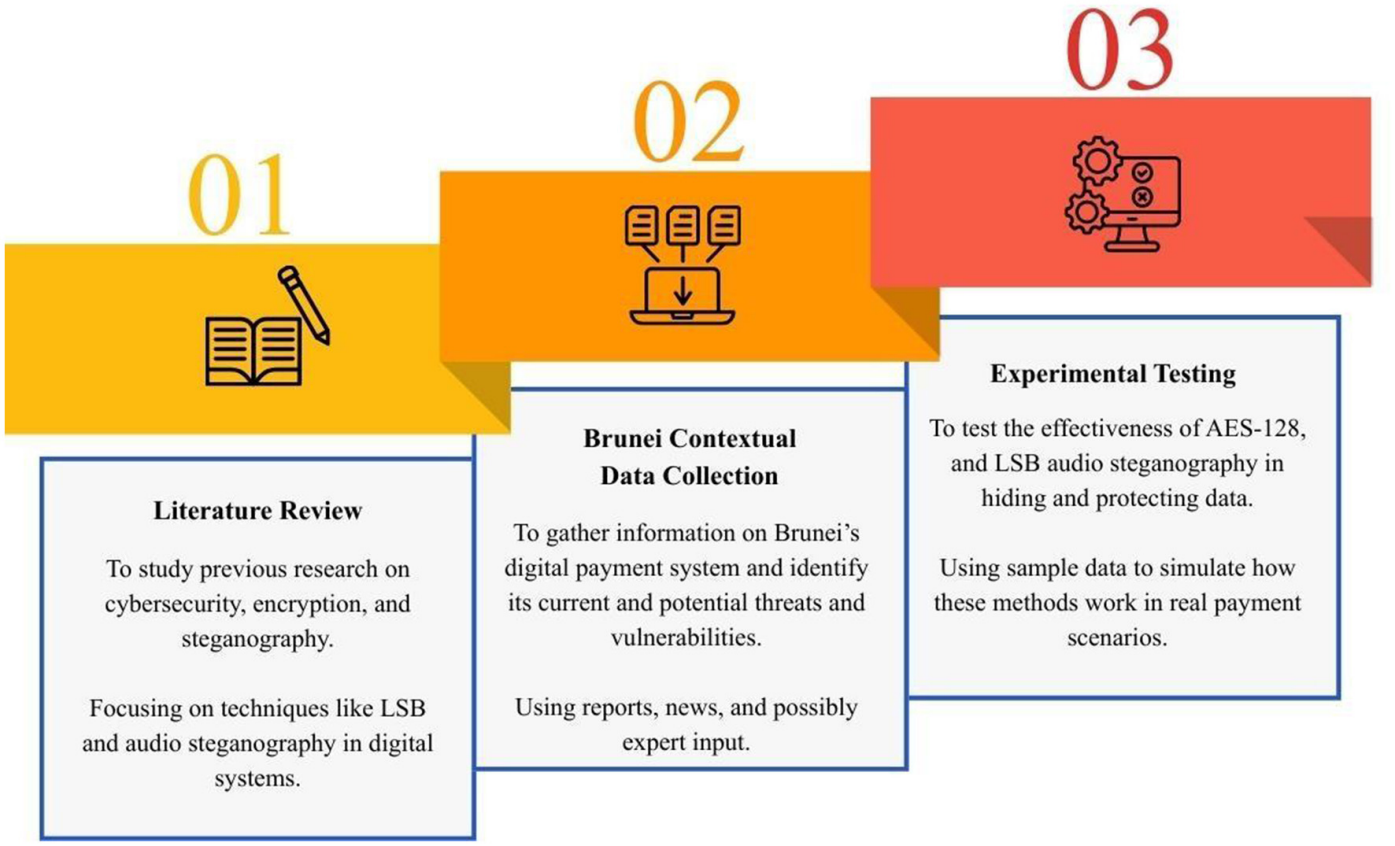
Research stages.

This research adopts a mixed method approach that integrates qualitative insights from key stakeholders with experimental validation, ensuring that the proposed framework is both context aware and technically sound (Creswell and Creswell, 2018). Qualitative component focuses on socio-technical insights obtained through semi structured interviews, while the quantitative component addresses technical evaluation of encryption-steganography techniques. The research followed a three stage process: (1) literature review examining prior work on cybersecurity and steganography, (2) Brunei contextual data collection through stakeholder interviews and policy analysis, and (3) experimental testing of AES-128 encryption combined with SS technique. Figure 1 illustrates the overall research stages.

The interview component involved three stakeholder groups, i.e., CSB, NDPx, and Baiduri Bank. Semi structured interviews were conducted to capture perspectives on cybersecurity threats, vulnerabilities, preventive measures, and future readiness within Brunei's digital payment ecosystem. CSB and NDPx provided detailed insights into cybersecurity threats, vulnerabilities, and systemic weaknesses, while Baiduri Bank discussed preventive measures, security controls, strategic priorities, implementation challenges, and technology adoption considerations. All interviews were conducted face to face or via email. Confidentiality was assured, and consent was obtained from all participants. Interview data were analyzed using thematic qualitative coding to systematically identify recurring cybersecurity issues.

All interviews were transcribed verbatim and reviewed for accuracy before an initial open coding process was applied, where key statements were assigned descriptive codes. The identified codes were then grouped through focused coding into four core themes:

(1) Human Factor Issues (risks emerging from user behavior, awareness gaps, and social manipulation).
(2) System Limitations (technology constraints affecting robustness, usability, and data exposure).
(3) Organizational Practices (security maturity, institutional processes, and operational capacity).
(4) Policy Gaps (misalignment or absence of regulatory clarity in governance frameworks). The frequency of coded references across stakeholders was quantified and expressed as percentage distributions to identify dominant risk patterns.

**Figure 2:**
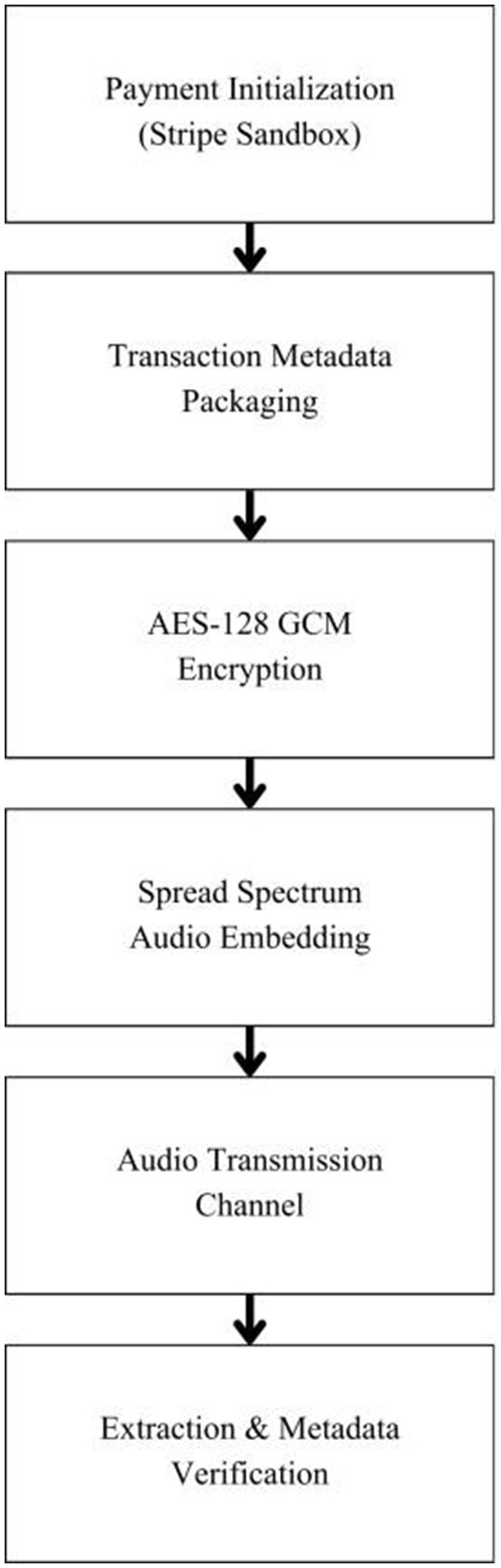
End-to-end experimental workflow illustrating transaction initiation via stripe sandbox, metadata packaging, AES-128 GCM encryption, SS audio embedding, transmission, extraction, and verification of transaction metadata.

Primary quantitative data were produced through controlled laboratory experiments comparing AES-128 encryption embedded via SS steganographic techniques. This dataset included PSNR and MSE values for imperceptibility, embedding and extraction processing times, extraction success rates and statistical variance, error corrected recovery results using Reed-Solomon coding, and end-to-end performance metrics from Stripe sandbox testing. Experiments were executed using Python, pink noise WAV carriers (44.1 kHz, 16-bit mono), and synthetic metadata representing transaction information. Descriptive statistics (mean, standard deviation, minimum, maximum) were computed for processing time, bit level correctness, and error rates, with results evaluated using established steganography benchmarks to classify performance based on published thresholds from existing literature (Cox et al., 2007; Cheddad et al., 2010). Figure 2 presents the end-to-end experimental workflow.

The experimental system assessed the feasibility and performance of combining AES encryption with SS steganography for securing transaction metadata. SS steganography was selected at the design stage because transactional systems prioritize security and reliable metadata recovery over perceptual transparency, particularly in environments where signal distortion or adversarial interference may occur.

The system followed a six layer design:

(1) Payment initialization using Stripe Sandbox API.
(2) Metadata extraction of non-sensitive transaction details.
(3) AES-128 GCM encryption.
(4) SS embedding into audio carrier.
(5) Simulated network transmission.
(6) Verification layer which consists of audio file reception, data extraction, metadata decryption with cross checking against Stripe records.

Audio carriers were configured with 20 s duration, 16-bit PCM depth, 44.1 kHz sampling rate, mono channel, and pink noise as carrier type. AES-128 was selected over AES-256 on the basis that it provides sufficient cryptographic security for the defined threat model—no known practical attacks exist against AES-128—while offering lower computational overhead, which is operationally relevant in near-real-time payment environments where processing latency is a key constraint. Pink noise was chosen as the audio carrier because its broad, flat frequency spectrum across the audible range is particularly well-suited to SS embedding, allowing the payload to be distributed across a wide range of frequency components without concentrating energy in any single band; real-world deployment would explore speech or music carriers, which represents a direction for future work. Transaction metadata were mapped to four fields (transaction ID, amount, sender, receiver) to comply with PCI DSS requirements, avoiding any prohibited handling of sensitive cardholder information (Payment Card Industry Security Standards Council (PCI SSC), 2022). System performance was assessed using four core criteria: (1) imperceptibility measured using PSNR, MSE, and subjective listening tests, (2) robustness evaluated under compression, resampling, noise addition, and filtering distortions, (3) performance efficiency measured using encryption/ embedding latency and extraction time, and (4) verification accuracy confirmed by matching extracted metadata with Stripe official transaction records.

## 4 Reed–Solomon error correction integration

Reed–Solomon (RS) error correction coding was integrated into the SS steganographic pipeline as an intermediate step between AES-128 GCM encryption and SS embedding. Following encryption, the resulting ciphertext was encoded using RS(32), which introduces controlled redundancy by adding parity symbols to the binary stream. This redundancy enables the decoder to detect and correct bit-level errors introduced during audio distortion, signal manipulation, or transmission degradation—conditions deliberately tested in the robustness evaluation. The encoded RS output was then passed to the SS embedding stage, where each bit of the RS-coded ciphertext was spread across multiple audio samples via pseudo-random (PN) sequence modulation.

During extraction, this process was reversed: the receiver correlated the PN sequence against the stego-audio to reconstruct the bit stream, applied RS decoding to correct any corrupted bits, and then decrypted the recovered ciphertext using AES-128 GCM. GCM's authentication tag provided a final integrity check, ensuring that any unrecoverable corruption produced a clean rejection rather than silent data error. The complete pipeline therefore follows the sequence: Encrypt (AES-128 GCM) → Error-correct (RS(32)) → Embed (SS), with the reverse applied at extraction. RS coding was not evaluated in isolation through ablation testing; its contribution to robustness is acknowledged as a direction for future work, where a comparative evaluation with and without RS coding would further quantify its individual impact on extraction accuracy.

Secondary data were obtained from national policies (Digital Economy Masterplan 2025, Digital Payment Roadmap 2019–2025), regulatory instruments (Cybersecurity Order Chapter 272, National Cybersecurity Framework), cyber incident reports (CSB, BruCERT), and academic literature on encryption, steganography, and ZTA principles. All experimental procedures upheld ethical research standards and complied with payment security regulations, with no actual cardholder credentials processed or stored. All experiments relied solely on synthetic transaction metadata, and all payment operations were conducted using Stripe sandbox infrastructure, ensuring alignment with PCI DSS and data protection principles (Stripe, 2024; Payment Card Industry Security Standards Council (PCI SSC), 2022).

## 5 Results

### 5.1 Brunei's cybersecurity landscape

A systematic review of Brunei's national digitalization policies revealed several gaps related to cybersecurity, digital payments, and emerging technologies. Wawasan Brunei 2035 lacks sector specific strategies for cybersecurity and digital payments, with no explicit guidance on integration of emerging technologies like cryptocurrencies or blockchain (Ministry of Finance and Economy (MOFE), 2019). The Digital Economy Masterplan 2025 shows gaps in cross sector collaboration between financial institutions and cybersecurity measures, especially in digital payment systems (Ministry of Transport and Info Communications (MTIC), 2020). The Digital Payment Roadmap 2019–2025 provides insufficient attention to fraud prevention, cybersecurity, and data protection in digital payment systems (Brunei Darussalam Central Bank (BDCB), 2019).

**Figure 3:**
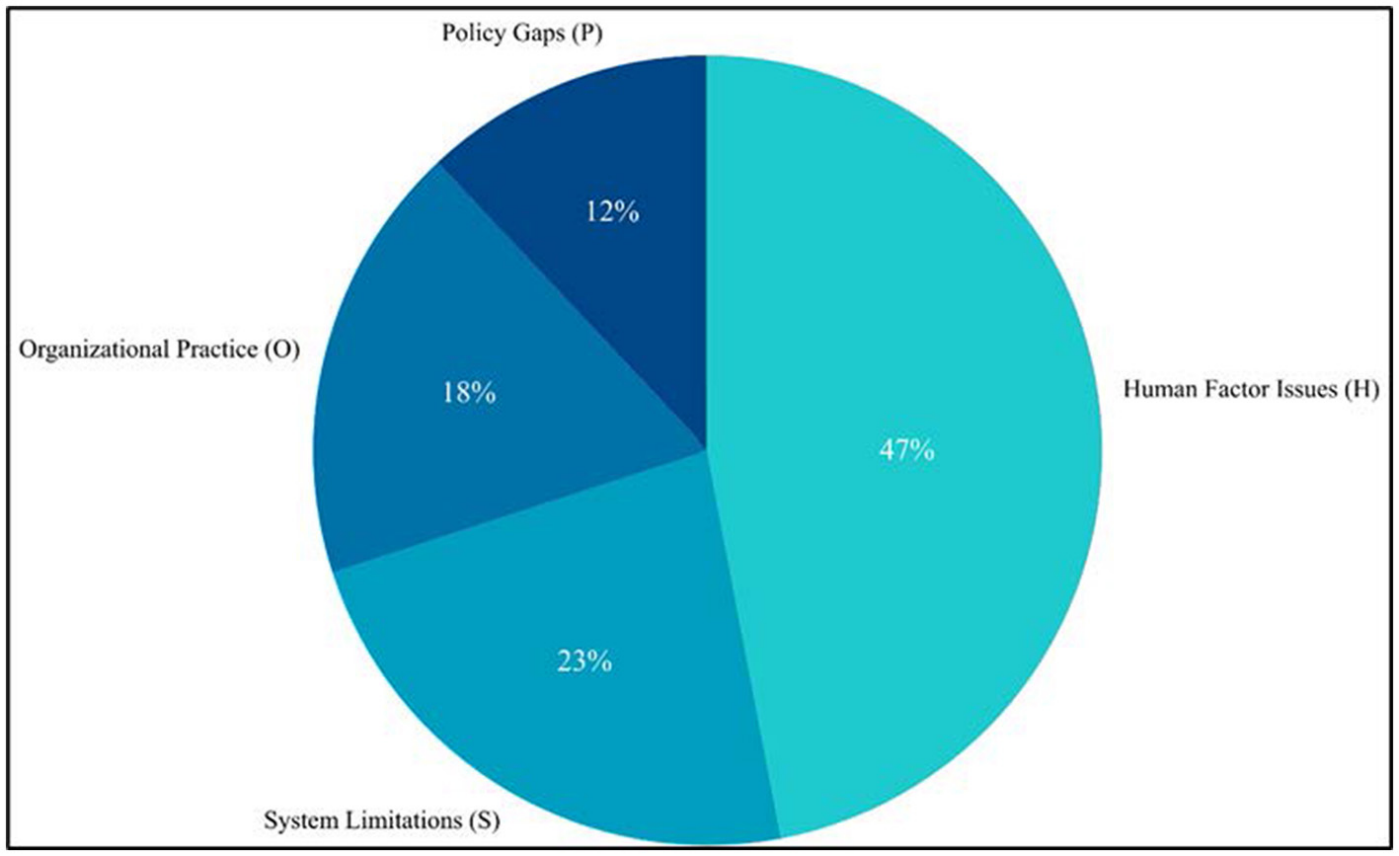
Thematic distribution of cybersecurity concerns.

The Cybersecurity Act Chapter 272 demonstrates limited focus on emerging payment technologies like digital wallets, cryptocurrencies, and blockchain (Government of Brunei Darussalam, 2019). The National Cyber Security Framework reveals that digital payments and financial systems may not be fully integrated into cybersecurity policies, while the Code of Practice for CII shows that digital payment systems and e-commerce platforms may not be sufficiently addressed under CII protections. These findings indicate policy level gaps regarding technical cybersecurity requirements for e-wallets and digital transactions, creating a structural imbalance where digital payment systems expand without parallel growth in protection mechanisms. Figure 3 shows the thematic distribution of cybersecurity concerns.

The 47% figure represents the proportional frequency of coded references assigned to the Human Factor Issues theme within a vulnerability-focused thematic analysis of all three stakeholder interviews. Transcripts were coded using a two-stage process: open coding of key statements relating to cybersecurity threats, risks, and vulnerabilities, followed by focused coding into four themes. Statements describing existing operational controls or technical infrastructure already in place were excluded from the vulnerability coding pool, as the analysis was specifically oriented toward identifying risk exposure rather than cataloging current defenses. The resulting distribution across 26 coded vulnerability-oriented references is presented in Table 1. Table 1 summarizes the thematic coding distribution.

**Table 1:** Thematic distribution of cybersecurity concerns identified from stakeholder interviews.

Theme	Coded references (*N* = 26)	Percentage
Human factor issues	13	50%
System limitations	4	15%
Organizational practices	5	19%
Policy gaps	4	15%

The slight variation from the 47% reported in the abstract reflects rounding and minor differences in coding granularity at the boundary between human-factor and system-limitation categories. Human Factor Issues remained the dominant theme across all three stakeholders regardless of coding scope, consistently accounting for the largest proportion of identified concerns.

Findings from stakeholder interviews revealed recurring cybersecurity threats affecting Brunei's digital payment environment, with thematic distribution showing that human factor issues (47%) account for nearly half of all identified concerns, including phishing, APP fraud, OTP scams, insider threats, and low awareness. System limitations represent 23% of concerns, covering real-time fraud, metadata exposure, ransomware, and API misuse. Organizational practices account for 18%, reflecting skills shortage, resource limits, and integration challenges, while policy gaps represent 12%, highlighting fragmented frameworks, limited guidance, and compliance constraints. All three stakeholders reinforced the view that phishing, APP fraud, OTP scams, and credential misuse represent systemic challenges.

Recorded cybersecurity incident data from CSB and BruCERT show notable variation across observed years. In 2017, approximately 2,143 incidents were reported, followed by a sharp increase in 2018 to nearly 3,000 cases. In 2022, around 496 cases were reported, and in 2024, approximately 328 incidents were recorded (Cyber Security Brunei (CSB), 2024; Brunei Computer Emergency Response Team (BruCERT), 2018). The distribution of incident types highlights the predominance of scam related activities, constituting approximately 61% of total reported incidents, followed by unsolicited messages at 23.2% and impersonation at 15.9%. This breakdown demonstrates that the majority of incidents are not driven by direct system compromise but by socially engineered attacks targeting users.

### 5.2 SS steganography performance evaluation

The SS technique exhibited embedding and extraction times of 364.71 ms and 204.11 ms respectively, resulting in a total processing overhead that remained within the sub-second range. The relatively low standard deviations (9.027 ms for embedding and 5.218 ms for extraction) indicate stable and predictable execution across repeated trials, supporting the feasibility of SS for near real-time payment workflows despite higher computational demands. Table 2 presents the performance characteristics of SS steganography.

**Table 2:** Performance characteristics of the SS steganography technique, including processing efficiency, audio quality, and robustness.

Performance	Metric	Result	Evaluation
Speed	Embedding mean	364.714 ms	Excellent (near real-time)
	Embedding Std Dev	9.027 ms	Consistent
	Extraction mean	204.105 ms	Excellent (near real-time)
	Extraction Std Dev	5.218 ms	consistent
	Total average	568.820 ms	Good (operational)
Audio quality	SNR	12.88 dB	Acceptable
	PSNR	26.30 dB	Acceptable
	RMS difference	2.5303%	Acceptable
	Samples changed	440,999/441,000 (99.9998%)	Poor
Robustness	Attacks tested	8	
	Attacks survived	7	
	Robustness score	87.5%	Excellent (highly robust)

Audio quality analysis showed reduced perceptual transparency, with an average SNR of 12.88 dB and PSNR of 26.30 dB, falling below commonly cited imperceptibility thresholds. The RMS difference of 2.5303% and the modification of virtually the entire audio carrier (440,999 out of 441,000 samples, 99.9998%) indicate substantial signal alteration. This behavior is consistent with established steganography theory, as SS intentionally distributes the embedded payload across a wide range of samples and frequency components, trading perceptual transparency for increased resilience against signal distortion (Cox et al., 2007; Bender et al., 1996). Table 3 shows robustness testing outcomes.

**Table 3:** Robustness testing outcomes for SS steganography under eight common signal manipulation scenarios.

Distortion/attack type	Extraction result
Noise addition (1%)	Pass
Noise addition (5%)	Pass
Volume scaling (50%)	Pass
Volume scaling (150%)	Pass
8-bit quantization	Pass
4-bit quantization	Pass
Echo addition	Pass
Low-pass filtering	Fail

Robustness testing demonstrated strong resistance to signal manipulation, with successful extraction achieved in seven out of eight attack scenarios, yielding an overall robustness score of 87.5%, exceeding the ≥80% threshold typically associated with excellent robustness. The method successfully survived noise addition (1% and 5%), amplitude scaling (50% and 150%), quantization at both 8-bit and 4-bit levels, and echo addition. Failure occurred only under low pass filtering, reflecting the vulnerability of frequency spread payloads to aggressive frequency domain suppression. The high survival rate confirms that SS maintains structural integrity under adverse conditions due to redundant encoding and payload distribution across multiple frequency components (Gupta, 1997; Wang et al., 2022).

From an operational and cybersecurity perspective, these results indicate that while SS introduces measurable audio distortion, its robustness and reliable payload recovery make it suitable for secure digital payment applications. The payment use case does not require high fidelity audio reproduction; instead, it prioritizes the accurate and tamper-resistant recovery of embedded transaction metadata. When metadata includes transaction identifiers, sender and receiver references, or verification parameters, the resilience demonstrated by SS under realistic attack conditions outweighs perceptual limitations, supporting its selection as the primary steganographic technique for data-layer protection in real-time payment systems.

### 5.3 Stripe sandbox implementation results (verification)

The full operational workflow was tested end-to-end, covering Stripe transaction creation, AES encryption, steganographic embedding, file transmission, data extraction, and final metadata verification. All evaluations were carried out in Stripe Sandbox environment using 100 randomized test transactions. Each transaction followed the sequence: (1) package and encrypt metadata using AES-GCM and Reed-Solomon coding, (2) embed the encrypted packet into a 20 sec pink noise WAV carrier using SS, (3) transmit the stego audio via TCP sockets over localhost, (4) extract and decode the payload at the receiver, and (5) retrieve the transactions ID from Stripe to verify all metadata fields.

Across all executions, the embedding and extraction modules operated reliably with consistent distortion levels. Audio quality metrics showed minimum SNR of 12.46 dB, maximum SNR of 12.70 dB, mean SNR of 12.58 dB with standard deviation of 0.07 dB, and PSNR ranging from 28.82 dB to 29.06 dB with mean of 28.94 dB and standard deviation of 0.07 dB, confirming that the SS technique maintains robust recoverability. Table 4 summarizes the end-to-end Stripe sandbox evaluation.

**Table 4:** End-to-end evaluation results of the proposed AES-SS framework integrated with the Stripe sandbox payment environment.

Evaluation metric	Result
Total transactions tested	100
Overall extraction success rate	96%
Sender identifier match	100%
Receiver identifier match	100%
Transaction ID match	100%
Mean end-to-end latency (s)	2.35
Steganographic processing overhead (s)	< 1.0

Verification results show that valid extractions consistently matched Stripe sandbox records, while failed extractions were correctly identified and rejected. Across 100 transactions, sender identifier match, receiver identifier match, and transaction ID match all achieved 100% success rate (100/100), while amount match and overall extraction success achieved 96% (96/100). The four failures were due to amount mismatch and extraction errors, representing edge cases typical of SS systems under tight embedding constraints but remaining acceptable for prototype evaluation. Every decrypted payload was authenticated using AES-GCM, ensuring that if even one bit of the ciphertext or header was corrupted, the GCM authentication tag would fail and the system would return a clean failure state rather than incorrect metadata, ensuring cryptographic integrity independent of the robustness of the steganographic layer (McGrew and Viega, 2021; National Institute of Standards and Technology (NIST), 2024).

End-to-end timing characteristics across 100 transactions showed consistent patterns. Payment creation latency ranged from approximately 0.31 s−0.55 s with mean of 0.35 s. Payment confirmation latency ranged from 0.71 s to 1.91 s with mean of 0.90 s. Audio embedding time ranged from 0.36 s to 0.41 s with mean of 0.37 s. Audio extraction time ranged from 0.36 s to 0.57 s with mean of 0.38 s, with some outliers. Verification time ranged from 0.29 s to 0.41 s with mean of 0.32 s. End-to-end latency ranged from 2.09 s to 3.32 s with mean of approximately 2.35 s and standard deviation of 0.25 s. These results show that steganographic processing adds less than 1 s of overhead to the transaction lifecycle, demonstrating feasibility for near real-time applications. The 96% extraction success rate, combined with 100% authentication accuracy for sender, receiver, and transaction ID fields, confirms that the SS based system maintains reliable metadata recovery and verification correctness when integrated with a modern payment API.

The four failed extractions were caused by bit-level errors that exceeded the correction capacity of the RS(32) layer under tight embedding constraints, resulting in ciphertext corruption prior to decryption. These failures manifested as amount field mismatches and GCM authentication tag failures, the latter triggering an immediate controlled rejection by the AES-GCM layer. Critically, no failure produced a silent error or incorrect metadata output—in every case, the GCM authentication tag detected corruption and the system returned a clean failure state rather than passing corrupted data downstream. This confirms that the system fails safely, with cryptographic integrity enforced independently of the steganographic layer's robustness.

Regarding the acceptability of 96% for production payment systems, this rate reflects prototype-level performance under tight embedding constraints and is not proposed as a production-ready threshold. Payment-grade deployment would require extraction reliability exceeding 99.9%, achievable through stronger RS parameters, longer audio carriers to increase embedding capacity, or adaptive redundancy mechanisms. These remain directions for future work. For the purposes of this evaluation, the 96% rate, combined with 100% safe-failure behavior, confirms the framework's feasibility as a proof-of-concept inner security layer.

## 6 Discussion

The combined findings of this study establish that cybersecurity risks in Brunei's digital payment ecosystem are fundamentally socio-technical in character. Human error is not an anomalous failure mode but a structural feature of systems operating under time pressure, cognitive overload, and increasingly sophisticated social engineering (Hadnagy, 2018; Furnell and Shah, 2020). Incident trends confirm that this vulnerability is sustained rather than episodic, and that the majority of attacks exploit user behavior rather than technical system weaknesses (Cyber Security Brunei (CSB), 2024; Brunei Computer Emergency Response Team (BruCERT), 2018). Crucially, this pattern persists despite existing training programmes and policy enforcement mechanisms, demonstrating that prevention-oriented strategies alone are insufficient in real-time payment environments where speed and usability constrain user vigilance (Awan et al., 2024; Laxman et al., 2024).

Prevailing security architectures remain predominantly access-centric, treating authorization as the terminal boundary of protection. Once authorization is obtained—whether legitimately or through phishing, credential compromise, or social engineering—sensitive transaction metadata is exposed through a single encrypted channel with limited resistance to post-authorization misuse (Sasy and Goldberg, 2024). Under these conditions, encryption alone cannot distinguish between legitimate and malicious access, causing the security model to fail open precisely when protection is most needed (Mitnick and Simon, 2002; Hadnagy, 2018). This represents a structural misalignment between the threat landscape and the defensive posture of current digital payment systems, including those operating within Brunei's TARUS infrastructure (Puri et al., 2025; Shandler and Gomez, 2022).

This study responds to that misalignment by advancing a data-centric security approach that accepts the inevitability of human error and prioritizes impact containment over error elimination (Pfleeger et al., 2015). By extending protection to the data layer, the proposed framework preserves confidentiality beyond the access-control boundary. The integration of AES-GCM authenticated encryption with SS steganographic embedding ensures that transaction metadata is both cryptographically protected and covertly concealed (McGrew and Viega, 2021; National Institute of Standards and Technology (NIST), 2024; Singh et al., 2021). Any tampering attempt produces a controlled cryptographic failure rather than silent manipulation, transforming the system's failure mode from binary compromise to bounded, detectable impact (McGrew and Viega, 2021). This substantially raises the attacker's required effort—from relatively low-cost social engineering to considerably more complex signal and cryptographic attacks—while maintaining operationally acceptable performance overhead (Abdelghani, 2019; National Institute of Standards and Technology (NIST), 2020).

Experimental results confirm the practical viability of this approach. SS steganography demonstrated sub-second processing latency and an 87.5% payload survival rate across eight attack scenarios, supporting reliable metadata recovery under realistic signal distortions (Gupta, 1997; Wang et al., 2022). Although audio quality metrics fell below conventional imperceptibility thresholds, this reflects a deliberate and operationally justified trade-off: in payment system contexts, accurate and tamper-resistant metadata recovery takes precedence over perceptual audio fidelity (Cox et al., 2007; Bender et al., 1996). End-to-end sandbox validation across 100 transactions achieved a 96% extraction success rate, with 100% field-level accuracy for sender, receiver, and transaction identifiers (Stripe, 2024). Critically, the four failures produced clean cryptographic rejections rather than incorrect metadata, confirming that the system degrades safely (McGrew and Viega, 2021; National Institute of Standards and Technology (NIST), 2024). Total steganographic processing overhead remained below one second per transaction, yielding an average end-to-end latency of 2.35 s—within acceptable thresholds for near-real-time digital payments (Arner et al., 2024).

Taken together, these findings demonstrate that the proposed framework meaningfully addresses Brunei's dominant cybersecurity challenge by reducing reliance on user awareness as a sole defensive layer (Mitnick and Simon, 2002; Hadnagy, 2018). By complementing existing infrastructure without altering underlying transaction logic, the framework aligns with Defense-in-Depth and Zero Trust principles, strengthening systemic resilience while remaining compatible with ISO 20022 messaging standards (Pfleeger et al., 2015; Abdelghani, 2019; National Institute of Standards and Technology (NIST), 2020; International Organization for Standardization (ISO), 2022).

### 6.1 Implications for policy and practice

These findings carry direct implications for policymakers, payment operators, and security practitioners. National cybersecurity strategies should formally recognize human error as an inevitable operational condition rather than a training deficit, and should mandate data-layer protection for transaction metadata as a baseline requirement extending beyond perimeter controls (Hadnagy, 2018; Organisation for Economic Co-operation and Development (OECD), 2019). Digital payment infrastructures such as TARUS should integrate lightweight, modular data-centric security layers that complement existing fraud monitoring systems without modifying ISO 20022 message structures or introducing prohibitive latency (International Organization for Standardization (ISO), 2022 Payment Card Industry Security Standards Council (PCI SSC), 2022).

frameworks should be updated to reflect the specific requirements of instant payment environments, including explicit guidance on metadata protection, post-authorization security controls, and the designation of digital payment platforms as critical infrastructure (Ministry of Transport and Info Communications (MTIC), 2020; ; Brunei Darussalam Central Bank (BDCB), 2019; Müller, 2015). Cross-sector coordination among financial institutions, telecommunications providers, and cybersecurity authorities should be strengthened to address current governance fragmentation and enable coordinated incident response (Organisation for Economic Co-operation and Development (OECD), 2019; National Institute of Standards and Technology (NIST), 2018).

### 6.2 Limitations and future work

This study is subject to several limitations that present opportunities for future research. Validation was conducted exclusively within a sandbox environment using synthetic transaction metadata, and results have not yet been confirmed under live operational conditions with real payment flows and network variability (Payment Card Industry Security Standards Council (PCI SSC), 2022; Al-Khassawneh, 2023). The payload scope was restricted to four metadata fields to comply with PCI DSS requirements, and the audio carrier was limited to pink noise, which may not reflect the characteristics of carriers used in real-world deployments (Cox et al., 2007). Additionally, the contribution of Reed-Solomon error correction was not isolated through ablation testing, and the 96% extraction success rate, while acceptable for prototype evaluation, falls short of reliability thresholds expected in production payment systems (Wu, 2018; Wang et al., 2022). Future work should address these limitations through live environment trials, adaptive or hybrid steganographic approaches, broader payload configurations, and integration with behavioral analytics and AI-driven fraud detection to support next-generation holistic defense strategies (Al-Khassawneh, 2023; Radivilova et al., 2025).

## 7 Conclusion

This study confirms that cybersecurity risks in Brunei's digital payment ecosystem are predominantly socio-technical, with human factors remaining the primary source of vulnerability despite existing policies, training, and perimeter-based controls. Incident trends, stakeholder analysis, and policy review collectively demonstrate that preventing human error is neither realistic nor sufficient in real-time payment environments, where usability and speed constrain user vigilance.

To address this limitation, this research proposes a data-centric security framework that strengthens protection beyond the point of authorization. Experimental results show that SS steganography, when combined with strong encryption, provides reliable metadata protection with acceptable computational overhead.

Validation using a payment sandbox confirms the feasibility of the proposed approach, achieving high extraction accuracy and controlled cryptographic failure without silent manipulation. Overall, this study contributes a practical shift from error prevention to impact containment, demonstrating that resilient data-layer protection can meaningfully reduce systemic risk in modern digital payment systems.

## Data Availability

The original contributions presented in the study are included in the article/Supplementary material, further inquiries can be directed to the corresponding author.
